# Factors in cognitive processing of Japanese loanwords by advanced Chinese Japanese-as-a-foreign-language learners

**DOI:** 10.3389/fpsyg.2023.1224830

**Published:** 2023-08-17

**Authors:** Yaoyao Geng, Qichao Song, Xiaodong Fei

**Affiliations:** ^1^Graduate School of Humanities and Social Sciences, Hiroshima University, Hiroshima, Japan; ^2^Beijing Center for Japanese Studies, Beijing Foreign Studies University, Beijing, China

**Keywords:** Japanese loanwords processing, English-Japanese phonological similarity, familiarity, context, English vocabulary proficiency, Chinese Japanese-as-a-foreign-language learners

## Abstract

**Introduction:**

Previous studies have highlighted the challenges faced by Chinese Japanese-as-a-foreign-language (JFL) learners (whose L2 is English) in acquiring L3 Japanese loanwords. These challenges arise from the linguistic characteristics of loanwords and the limited emphasis on teaching and learning them. However, there is a lack of research on the specific factors that influence the processing of Japanese loanwords among Chinese JFL learners. Significant motivation exists, therefore, to investigate these influencing factors as they provide valuable insight into the integration of phonographic and ideographic language systems, ultimately facilitating future lexical acquisition.

**Methods:**

In this study, an experiment was conducted on 31 Chinese JFL learners to investigate the effects of loanword familiarity, English vocabulary proficiency, English-Japanese phonological similarity, and context on the processing of Japanese loanwords.

**Results:**

Data analysis, using a (generalized) linear mixed-effect model, provided the following insights: (1) the processing of Japanese loanwords is influenced by English-Japanese phonological similarity, loanword familiarity, context, and learner English proficiency. Among these four factors, familiarity has the most significant impact on Japanese loanword processing; (2) the effects of context and phonological similarity on the processing of Japanese loanwords are not consistently positive. As learners improve their proficiency in L3 Japanese, they tend to decrease their reliance on English knowledge and instead access loanword representations directly to conceptual representations.

**Discussion:**

Based on the findings of this study, a processing model for Japanese loanwords among advanced Chinese JFL learners is proposed. The model emphasizes the critical importance of the characteristics of loanwords, including phonological similarity and familiarity. It is necessary to determine the specific circumstances in which context considerably enhances learner processing ability.

## 1. Introduction

Lexical processing in second languages (L2s), mainly in phonographic languages, has received considerable attention lately. Understanding how L2 learners store and retrieve words provides valuable insight into lexical acquisition and processing (Lee et al., 2018; Jankowiak, 2021). In recent years, there has been a surge in studies focusing on the processing of Japanese *Kanji* words by Chinese Japanese-as-a-foreign-language (JFL) learners. This has provided fresh empirical evidence in the field of ideographic writing systems (e.g., Mori, 2014; Fei and Li 2017; Fei et al., 2022; Song et al., 2023). Japanese writing systems can be divided into three types: *Kanji, Hiragana, and Katakana*. These systems encompass both phonographic and ideographic elements. *Kanji* originated from Chinese characters and is used by both Chinese and Japanese. *Hiragana* is used for native Japanese words and grammatical elements, whereas *Katakana* is primarily used for loanwords, which are commonly referred to as 外来語 “*Gairaigo*” or カタカナ語 “*Katakanago*” (Kess and Miyamoto, 2000). With the rapid advancement of informatization and globalization, the number of Japanese loanwords is increasing. According to Sube (2013), approximately 80 percent of the loanwords listed in the Iwanami Kokugo Jiten (Iwanami Japanese Dictionary, 3rd Edition) are derived from English. In China, there are millions of Japanese language learners, they are number second only to the number of English learners. Investigating the factors that influence the processing of loanwords by Chinese JFL learners can provide valuable insight into the integration of phonographic and ideographic language systems for future lexical acquisition research.

Lexical proficiency encompasses two dimensions: vocabulary breadth and vocabulary depth (Wesche and Paribakht, 1996; Li and Kirby, 2015), which relate to the relationship between quantity and quality. As learners progress to an advanced stage in their language-learning journey, when their vocabulary breadth reaches a certain level, how quickly they process and retrieve existing vocabulary from their mental lexicon becomes increasingly important. Therefore, it is necessary to explore the factors that affect the lexical processing which facilitates this process. Nevertheless, as loanwords are an important component of Japanese vocabulary, little research has been conducted on the processing of loanwords by Chinese JFL learners compared with research on Japanese *Kanji* word processing (Tamaoka, 1997; Yamato et al., 2010; Yamato and Tamaoka, 2011, 2013; Jha et al., 2018; Tamaoka, 2018).

To fill this research gap, the present study investigates factors that influence the processing of loanwords by advanced Chinese JFL learners. It examines the influence of English-Japanese phonological similarity, familiarity, context, and English vocabulary proficiency on the processing of Japanese loanwords.

## 2. Literature review

### 2.1. Hypotheses on an L2 lexical processing model

Numerous studies have provided substantial evidence supporting the phenomenon of “non-selective processing” in bilinguals. Results suggest that, when processing one language, bilinguals unintentionally activate both the conceptual and lexical representations of another (Hermans et al., 1998; De Groot et al., 2000; Van Hell and Dijkstra, 2002; Singh et al., 2014; Dijkstra and Walter, 2018). The effects of orthographic, phonological, and semantic similarities have been widely documented in both first language (native language, L1) and L2 contexts (Antón and Duñabeitia, 2020). Nonetheless, whether these effects also exist when processing L2s and L3s simultaneously is unclear.

Previous studies (e.g., Dewaele, 1998; De Angelis and Selinker, 2001; Jasone, 2001) have indicated that the language knowledge acquired in one language can affect the processing of another language, particularly in the case of L2 learners. Chinese JFL learners possess knowledge not only of their own Chinese (L1), but also of English (L2), which they typically study in school to prepare for university entrance exams. As a result, Chinese JFL learners are likely influenced by their knowledge of English when acquiring Japanese (L3). Additionally, the Japanese language incorporates a considerable number of loanwords derived from English, which are written in *Katakana* using English pronunciation. This means that there are numerous English-derived loanwords that exhibit high phonological similarity to English, enabling Chinese JFL learners to draw on their English knowledge when processing Japanese loanwords (Hoshino and Kroll, 2008; Yamato and Tamaoka, 2013). Therefore, investigating the impact of English (L2) on the processing of Japanese (L3) loanwords by Chinese JFL learners offers a valuable approach to understanding the interaction between languages and their influence on language processing.

**Figure 1:**
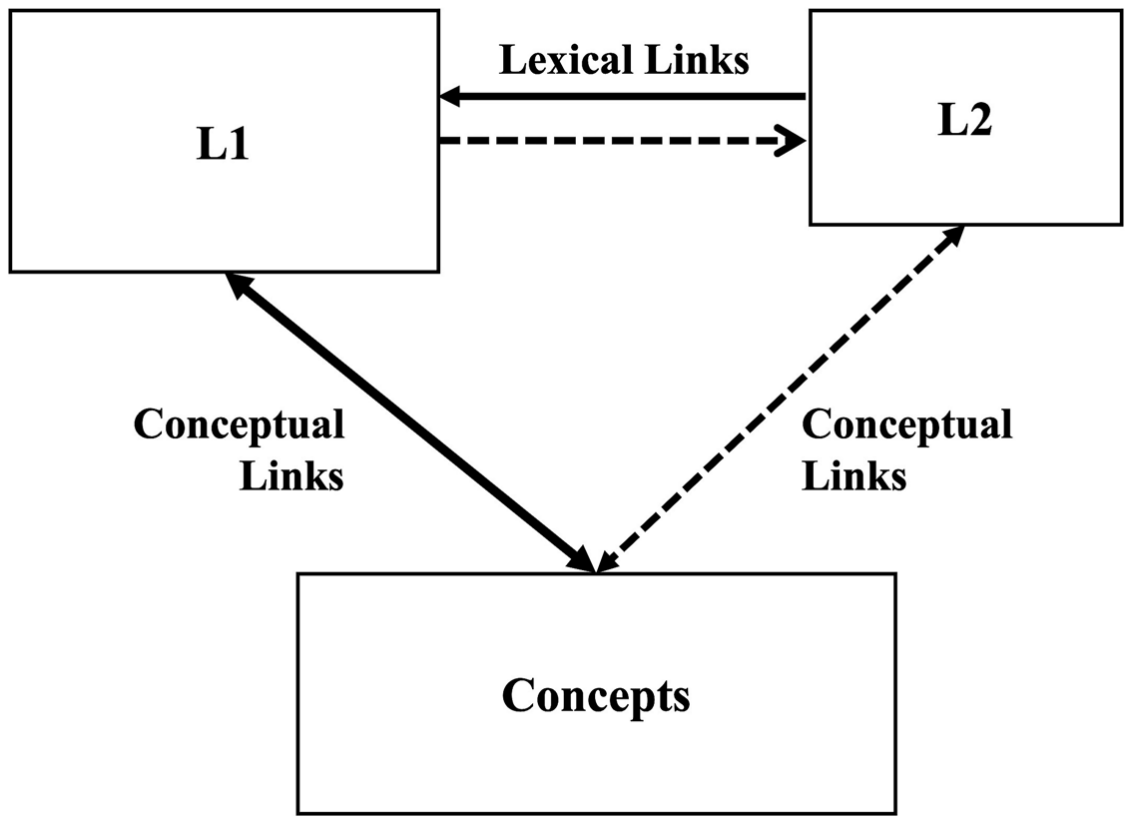
Revised hierarchical model (Kroll and Steward, 1994).

The Revised Hierarchical Model (Figure 1) proposed by Kroll and Steward (1994) has been widely employed in bilingual lexical processing research (e.g., Silverberg and Samuel, 2004; Ferré et al., 2006; Kroll et al., 2010). According to this model, links between lexical and conceptual representations in a target language are believed to develop as learner proficiency in the target language improves. As L2 proficiency increases, reliance on the L1 for conceptual links gradually diminishes, particularly when dealing with two languages from different language families. Numerous empirical studies have been conducted on *Kanji* word processing among Chinese JFL learners (e.g., Matsumi et al., 2012; Fei et al., 2022; Song et al., 2023). During the initial stages of Japanese lexical acquisition, links between L3 Japanese lexical and conceptual representations tend to be weak. Therefore, the processing of *Kanji* word is expected to rely on translation equivalents in the learner’s L1 (i.e., Chinese), which serves as a bridge to accessing corresponding conceptual representations. In contrast, an advanced learner’s links between Japanese lexical and conceptual representations tend to be strong, facilitating his or her direct access to conceptual representations without relying heavily on L1 knowledge. Japanese (L3) loanwords are related semantically to the Chinese (L1), and phonetically to English, the acquired L2. Therefore, unlike Chinese-Japanese bilingual processing in *Kanji* words, processing models of loanwords may involve a complex trilingual interaction in terms of phonetics and semantics. Nonetheless, as mentioned above, there is limited research and theoretical discussion on the construction of such processing models and their theoretical implications.

### 2.2. Factors affecting the processing of Japanese loanwords

Previous research has focused on the processing of Japanese loanwords in the context of bilingualism, specifically examining how it is influenced by English-Japanese phonological similarity and learners’ English proficiency (Yamato and Tamaoka, 2013; Tamaoka, 2018). Moreover, studies have highlighted the significance of other influencing factors, including familiarity and the presence or absence of context, in shaping the processing of Japanese loanwords (Geng, 2022).

As mentioned earlier, the processing of loanwords is influenced by English-Japanese phonological similarity and the English proficiency of learners because many Japanese loanwords are derived from English. In contrast to English syllables, which display considerable structural variation and can be quite complex, there are only four fundamental syllable structures in Japanese: (C)V (C = consonant; V = vowel), (C) VV, (C) VN (N = nasal), and (C) VQ (Q = first part of a geminate obstruent) (Tajima et al., 2002). Therefore, loanwords undergo phonological assimilation to conform to the primarily CV-based structure of Japanese. For example, “テキスト,” borrowed from the word “text,” is pronounced as /tekisuto/, and “ドリーム,” borrowed from the word “dream” is pronounced as /dori:mu/. Tamaoka (2018) conducted a priming experiment and found that loanword processing was facilitated when there was a high degree of phonological similarity with English. Tamaoka (1997) also found that, compared with those of English JFL learners, the accuracy rates of Chinese JFL learners were significantly lower. This suggests that English vocabulary proficiency plays an important role in accurately processing Japanese loanwords. Yamato and Tamaoka (2013) further investigated the influence of English knowledge on the processing of loanwords. They revealed that English proficiency, as an L2, significantly influenced the processing of loanwords in Japanese as an L3. However, the Japanese proficiency levels of the participants of Yamato and Tamaoka (2013) range from intermediate to advanced. For Chinese learners, the level of English-Japanese phonological similarity is directly related to the number of English and Japanese words stored in their mental lexicon. Therefore, when examining the impact of English on the processing of Japanese loanwords, it is crucial to consider both phonological similarity and L2 and L3 proficiency. However, the research described above does not provide an in-depth exploration of the relationship between the influence of phonological similarity and language proficiency.

Research has consistently demonstrated the significant role of context in L2 lexical processing (Stanovich and Richard, 1983; Balota et al., 1985; Sereno et al., 2003; Goldwater et al., 2009; Perea et al., 2013). A recent study conducted by Song and Fei (2022) highlighted the substantial impact of context on the processing of Japanese vocabulary by Chinese JFL learners. Nevertheless, the role of context in facilitating loanword comprehension has not been confirmed. Jha et al. (2018) discussed the processing of novel words written in *Katakana* by advanced Chinese JFL learners and found no such significant role involving context. However, it is important to note that the stimuli used in Jha et al. (2018) were non-words written in *Katakana*, which may have caused participants to focus more on processing the meaning of the target non-words rather than comprehending the sentence as a whole. Thus, further investigation is necessary to gain a clearer understanding of how context influences the processing of loanwords by Chinese JFL learners. Familiarity is also an important factor in influencing a learner’s processing of Japanese loanwords. Yamato and Tamaoka (2011) found that for Chinese JFL learners with low proficiency in Japanese, low-familiarity loanwords significantly influenced processing speed. Yamato et al. (2010) suggested that, for Chinese JFL learners, the processing efficiency of loanwords was influenced to a large extent by learning duration. Specifically, as the learning duration increases, their familiarity with Japanese loanwords also improves. Consequently, the activation threshold for words decreased, allowing for faster activation of representations. Additionally, a previous study has revealed that, in advanced Chinese EFL (English-as-a-foreign-language) learners, processing patterns are still influenced by English word familiarity (Li et al., 2011). This suggests that, if bilingual individuals are highly familiar with L2 vocabulary, they can directly access concepts from the L2 (Chen and Leung, 1989). Consequently, further research is needed to explore the influence of familiarity on the processing of loanwords among Chinese JFL learners as their Japanese proficiency improves.

In summary, there is currently a lack of research on the various factors that influence the processing of Japanese loanwords. Existing studies have focused on bilingual proficiency, phonological similarity, and context, without fully examining their interactions and specifically emphasizing advanced Chinese JFL learners. Therefore, research on the influence of these factors is urgently required to elucidate this phenomenon.

### 2.3. Objectives and hypotheses of this study

This study investigates the influence of English-Japanese phonological similarity, familiarity, context, and English vocabulary proficiency on the processing of Japanese loanwords in advanced Chinese learners. Motivation for the study rests on the lack of comprehensive research that systematically investigates the various influencing factors, and the learner’s Japanese proficiency not being restricted to one level only. Whether such influencing factors undergo changes during processing with increasing learner language proficiency is investigated. This study focuses on the following questions:

RQ1: How do English-Japanese phonological similarity, familiarity, context, and English vocabulary proficiency influence the accuracy rates and reaction times of loanword processing among advanced Chinese JFL learners?

RQ2: What lexical processing models do advanced Chinese JFL learners utilize when processing Japanese loanwords?

Based on the review of existing studies given above, the hypotheses of this study are as follows:

*H1*: Previous research has demonstrated that phonological similarity enables Chinese JFL learners to rely on the pronunciation of corresponding English words in their mental lexicon (Yamato and Tamaoka, 2013; Tamaoka, 2018), facilitating the processing of Japanese loanwords. The current study expects English-Japanese phonological similarity to promote the processing of Japanese loanwords.

*H2*: Previous research (Li et al., 2011) shows that even advanced learner processing is influenced by familiarity. Therefore, it is speculated that, in the processing of Japanese loanwords by advanced Chinese JFL learners, both the accuracy rates and reaction times will be positively influenced by familiarity with Japanese loanwords.

*H3*: Concerning context, although Jha et al. (2018) found that the presence or absence of context did not influence the inference of unknown loanword meanings, considering their experimental materials were non-words and considering the numerous findings from previous research on language processing that demonstrate the facilitating role of context in lexical processing, it is speculated that context can play a facilitating role (e.g., Sereno et al., 2003; Goldwater et al., 2009). Specifically, we anticipate that both the accuracy rates and reaction times will improve.

*H4*: Previous research has confirmed that a high English vocabulary proficiency is associated with highly efficient processing of Japanese loanwords (Tamaoka, 1997; Yamato and Tamaoka, 2013). Therefore, it is speculated that English vocabulary proficiency may facilitate the processing of Japanese loanwords.

## 3. Materials and methods

### 3.1. Participants

An experiment was conducted on 31 advanced Chinese JFL learners, comprising 19 females and 12 males, with ages ranging from 22 to 26 years old. The participants had a mean Japanese study time of 6.02 (*SD* = 1.42) years. And all were enrolled in the same graduate school in China, majoring in Japanese language and literature. They began studying Japanese in their first year of college and passed the Japanese-Language Proficiency Test (JLPT) at the N1 level (the highest level, which, according to JLPT’s official instructions, means they obtained the ability to understand Japanese in various circumstances). All participants had normal vision (with corrected vision). The participants, therefore, belonged to a homogeneous group of learners. We provided them with the Language History Questionnaire (Li et al., 2020) to assess the participants’ proficiency and usage time in Chinese, Japanese, and English. Analysis of the questionnaire responses revealed that all participants were unbalanced trilinguals, with their highest proficiency in Chinese, followed by Japanese and English [*Fs* (2, 60) = 88.23–193.90, *ps* < 0.001, see Table 1].

**Table 1:** Participants’ self-reported language proficiency and comparisons between Chinese, Japanese, and English.

	C	J	E	Comparison
L	6.71 (0.53)	4.90 (0.70)	3.19 (0.95)	C > J > E^***^
S	6.42 (0.85)	4.65 (0.84)	2.77 (1.20)	C > J > E^***^
R	6.58 (0.96)	5.58 (0.99)	3.81 (1.38)	C > J > E^***^
W	6.16 (1.13)	4.81 (1.01)	2.90 (1.22)	C > J > E^***^
Time of Usage (h/day)	11.78 (3.96)	5.98 (3.45)	1.02 (0.94)	C > J > E^***^

*** *p* < 0.001; L, listening; S, speaking; R, reading; W, writing; C, Chinese; J, Japanese; E, English.

*Tukey’s HSD tests* were used for multiple comparisons.

### 3.2. Design

In this study, the impact of four independent variables on the accuracy rate and reaction time of Chinese JFL learners was examined. These variables were English-Japanese phonological similarity, familiarity with loanwords, context and English vocabulary proficiency.

### 3.3. Materials

Forty-four word items and 22 sentences for contextual condition (see supplementary materials) were created. The selection of loanwords was based on the list of Basic Loanwords List by Mochizuki (2012) and Japanese textbooks (Peng and Moriya, 2007) used in Chinese universities. To ensure an appropriate level of difficulty in the loanword materials, we utilized “Reading Tutor,^1^” a widely recognized website for Japanese education research that assesses the difficulty of Japanese content. The difficulty of the loanwords was adjusted based on the analysis results from “Reading Tutor,” resulting in a word list consisting of 90 English-derived loanwords. The control procedures for the various indicators of the experimental materials are described as follows.

[Phonological Similarity] The 90 loanwords were recorded in both standard English and Japanese by a native English speaker from England and a native Japanese speaker from Japan. Due to the relatively high familiarity of Japanese loanwords among Chinese JFL learners, especially among advanced learners, there may have been a bias in their perception of the phonological similarity between English and Japanese loanwords. Therefore, to specifically examine the phonological similarity for Chinese learners, we recruited 20 Chinese university students who had prior English learning experience and achieved level 4 in the College English Test, a well-known English proficiency test held in China annually to test the English proficiency of Chinese university students. These participants had no prior experience in learning Japanese. They were assigned a phonological similarity judgment task using a seven-point rating questionnaire. The rating scale ranged from 1 (not similar at all) to 7 (very similar).

[Familiarity] For the familiarity evaluation of the selected materials, we recruited 76 Chinese JFL learners who had a background in Japanese learning similar to that of the participants in the experiment. They were instructed to rate the materials on a seven-point scale, ranging from 1 (not familiar at all) to 7 (very familiar). The reason we used a seven-point rating questionnaire rather than a five-point one was because phonological similarity and familiarity were used as continuous variables when conducting data analysis. Therefore it is ideal to have sufficient statistical dispersion to ensure a more significant linear change between familiarity and phonological similarity judgment values.

[Context] To ensure differentiation between high and low phonological similarity and familiarity in the experimental materials, we carefully selected 44 loanwords from the initial pool of 90. The selection was based on the evaluation results of phonological similarity and familiarity obtained from previous assessments. These loanwords were then equally divided into two groups, an isolated condition group and a contextual condition group, with 22 words assigned to each condition. During the selection process, we took into account various factors, such as word frequency (based on the Balanced Corpus of Contemporary Written Japanese, BCCWJ), mora number, phonological similarity, and familiarity, and ensured that there were no significant differences in the above indicators between the two sets of materials [*ts* (42) = 0.06–1.33, *ps* > 0.192, see Table 2].

**Table 2:** Summary of characteristics of the test items.

	Mora count	Logged-transformed frequency	Phonological similarity	Familiarity	Examples
Isolated condition	3.45 (0.80)	3.16 (0.51)	4.25 (1.67)	5.81 (0.97)	プラス (plus)
Contextual condition	3.86 (1.21)	3.06 (0.52)	4.28 (1.70)	5.89 (1.30)	台風のせいでテレビのアンテナが折れてしまいました。(Due to the typhoon, the TV antenna got broken)

Results are expressed as mean (*SD*).

Contextual sentences containing the target loanwords were carefully selected from the BCCWJ and online sources. The selected contexts were then evaluated in terms of their degree of matching with the loanwords. To ensure the highest level of relevance between the contextual materials and loanwords, we invited five advanced Chinese JFL learners to reconfirm the suitability of the chosen contexts. Additionally, we utilized the “Reading Tutor” tool to evaluate the level of difficulty of the materials used in our experiment, ensuring that the chosen contexts did not hinder participants’ smooth reading. The results from the “Reading Tutor” indicated that all sentences were considered “very easy.” Therefore, the level of difficulty of the contexts did not impact participant processing.

[English Vocabulary Proficiency] To assess the participants’ English vocabulary proficiency, we utilized the Bilingual Version of Vocabulary Size Test (VST) developed by Nation and Beglar (2007). This modified version of the Vocabulary Level Test was created based on Nation (1983). The VST focuses on evaluating learners’ receptive vocabulary and consists of 14 sections, each representing a different vocabulary level ranging from 1,000 to 14,000 words. Each section comprises 10 multiple-choice questions, featuring an English word, a contextless sentence, and four Chinese semantic alternatives. Participants are required to choose the correct answer from the four options, and they receive one point for each correct response. This test has been widely used to assess the English writing vocabulary of L2 learners due to its reliability and validity (Nation and Beglar, 2007).

[Fillers] In addition to the 44 selected words for the lexical judgment task, 28 non-words (14 for the isolated condition group and 14 for the contextual condition group) as fillers were selected. These non-words consisted of two types: those that were similar to the original words, such as “バイオリーン” (correct loanword: “バイオリン,” violin), and those that were dissimilar, such as “サドバハヤ.” The sentences used for fillers in the contextual condition group were collected from the BCCWJ and Japanese textbooks mentioned above. The “Reading Tutor” showed that all sentences were “very easy.”

### 3.4. Apparatus

A personal computer (SOTEC N15 WMT02) was used for the loanwords’ presentation. The experimental program was created using SuperLab Pro 4.0 (Cedrus Corporation).

### 3.5. Procedure

**Figure 2:**
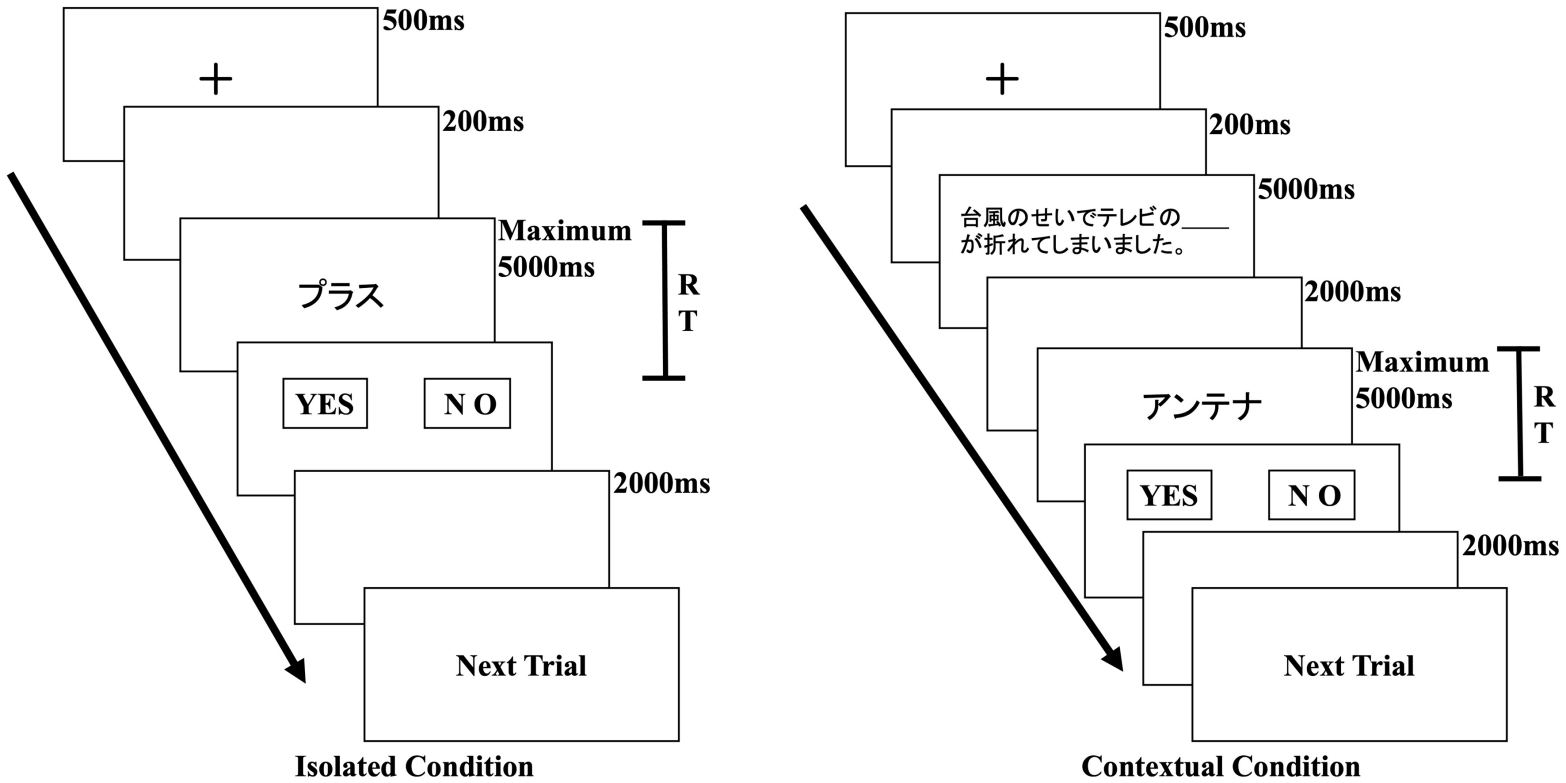
Flow of one experimental trial under isolated and contextual conditions.

Because of the potential limitations associated with using reaction time as a measure in psychological experiments (see Crocetta and Andrade, 2015), we carefully selected the experimental apparatus and created a controlled environment for implementation. Participants were tested individually in a sound-attenuated room. Figure 2 shows the experimental procedure for one trial under both isolated and contextual conditions. Under the isolated condition, each trial began with the display of a fixation point on the screen for 500 ms, indicating the upcoming appearance of a loanword. After a 200 ms blank interval, a loanword was presented on the screen. The maximum presentation time for each word was 5,000 ms. Once a participant responded, or if 5,000 ms elapsed without a response, the next trial commenced after 2000 ms. The stimuli were presented in randomized order. In the contextual condition group, a sentence was presented before a loanword was presented. The sentence contained a blank space, and participants were instructed to read and understand the sentence and judge whether the upcoming loanword existed in Japanese. Except for the inclusion of sentences before participants make judgments, the procedure for presenting stimuli was the same in both the isolated and contextual condition groups. Participants were given instructions to determine, as quickly and accurately as possible, whether each stimulus constituted a genuine word. Before the formal experiment began, we conducted sufficient practice and testing sessions to ensure that the participants fully understood the experimental task.

## 4. Results

### 4.1. Data manipulation

We excluded 23 out of the trials whose reaction times were longer than 3,500 ms and ± 2.5 *SD*s above and below the mean. The percentage of exclusion was 1.70%. To deal with skewed data, reaction times were log-transformed. The phonological similarity and English vocabulary proficiency data were standardized. Data analyses were conducted using the software R (version 4.2.1, R Core Team, 2022). We used linear-mixed-effect model with the lme4 (Bates et al., 2015) and lmerTest (Kuznetsova et al., 2017) packages. The model with the lowest Akaike information criterion (AIC) was selected as the optimal model for model fitting. The software jamovi (version 2.3, jamovi project, 2022) was used to examine interactions. The Wald *z*-distribution was used to compute *p*-values for the accuracy rates data. The Wald *t*-distribution approximation was used to compute *p*-values for the reaction times data.

### 4.2. Results of the accuracy rates data

Using the AIC, familiarity, context, phonological similarity, first-order interaction of similarity and context, and second-order interaction of similarity, the context and familiarity were selected as fixed effects, and participants and items were selected as random effects in the model. The results for the accuracy rates are shown in Table 3. The main effect of familiarity was significant (*χ*^2^ (1) = 4.45, *p* = 0.035), indicating that the accuracy rate increased with increasing familiarity. The main effect of phonological similarity was significant (*χ*^2^ (1) = 4.91, *p* = 0.027), indicating that the accuracy rate increased with increasing phonological similarity. The main effect of context was not significant (*χ*^2^ (1) = 1.43, *p* = 0.231).

**Table 3:** Results of GLME model analysis of accuracy rates.

Variables	Estimate	SE	*z*	*pr* (*>|z|*)
Intercept	9.22	2.48	3.72	<0.001
Familiarity	3.19	1.51	2.11^*^	0.035
Phonological Similarity	3.06	1.38	2.22^*^	0.027
ContextY	−3.51	2.93	−1.20	0.231
Phonological Similarity: ContextY	−9.24	3.09	−2.99^**^	0.003
Familiarity: Phonological Similarity: ContextN	1.26	1.11	1.14	0.256
Familiarity: Phonological Similarity: ContextY	−7.03	1.74	−4.04^***^	<0.001

^*^p < 0.05, ^**^p < 0.01, ^***^p < 0.001.

Participants = 31. Items = 44. Total observation = 1181.

SE, standard error. *df*, degree of freedom.

The optimal model is *glmer* [acc ~ Familiarity + Phonological Similarity + Context + Phonological Similarity: Context + Familiarity: Phonological similarity: Context + (1|item) + (1|participant), data = data, glmerControl optimizer = “bobyqa,” optCtrl = list (maxfun = 100000)].

Given the significant first-order interaction between phonological similarity and context (*χ*^2^ (1) = 8.95, *p* = 0.003), simple main effects were analyzed (Table 4). The results indicate that, under the isolated condition, loanwords with high phonological similarity had significantly higher accuracy rates than those with low phonological similarity (*z* = 2.67, *p* = 0.008). In contrast, under the contextual condition, loanwords with high phonological similarity had lower accuracy than those with low phonological similarity (*z* = −2.72, *p* = 0.007). Additionally, when phonological similarity was low, accuracy tended to be higher with context than without context (*z* = −1.76, *p* = 0.078). However, when phonological similarity was high, accuracy was significantly lower with context than without context (*z* = 2.51, *p* = 0.012).

**Table 4:** Results of simple main effects between context and phonological similarity.

Moderator Levels	Contrast	Estimate	SE	*z*	*pr* (*>|z|*)
N	High – Low	7.69	2.88	2.67^**^	0.008
Y	High – Low	−1.56	0.57	−2.72^**^	0.007
Low	N – Y	−5.73	3.25	−1.76^†^	0.078
High	N – Y	12.76	5.07	2.51^*^	0.012

† *p* < 0.10, ^*^p < 0.05, ^**^p < 0.01.

N, isolated condition. Y, contextual condition.

SE, standard error; *df*, degree of freedom.

High, Mean + *1SD*. Low, Mean-*1SD*.

Furthermore, there was a significant second-order interaction between context, phonological similarity, and familiarity (*χ*^2^ (2) = 17.09, *p* < 0.001). The results for the simple main effects are shown in Table 5. When both familiarity and phonological similarity are high, the context has an inhibitory effect. In contrast, when both familiarity and phonological similarity are low, despite the relatively low accuracy rates in the presence of context, there is not a significant effect from the context.

**Table 5:** Results of simple main effects between context, phonological similarity, and familiarity.

Moderator Levels						
**Phonological similarity**	**Familiarity**	**Contrast**	**Estimate**	**SE**	***z***	***pr* (*>|z|*)**
Low	Low	N – Y	2.55	2.94	0.87	0.385
	High	N – Y	−14.02	4.96	−2.82^**^	0.005
High	Low	N – Y	4.47	3.79	1.18	0.239
	High	N – Y	21.04	7.02	3.00^**^	0.003
Moderator levels						
**Phonological similarity**	**Context**	**Contrast**	**Estimate**	**SE**	***z***	***pr (>|z|)***
Low	N	High – Low	1.93	1.45	1.33	0.183
	Y	High – Low	10.22	2.84	3.60^***^	< 0.001
High	N	High – Low	4.45	2.22	2.00^*^	0.045
	Y	High – Low	−3.84	1.60	−2.40^*^	0.016
Moderator Levels						
**Familiarity**	**Context**	**Contrast**	**Estimate**	**SE**	***z***	***pr* (*>|z|*)**
Low	N	High – Low	6.43	1.87	3.43^***^	< 0.001
	Y	High – Low	5.47	1.47	3.73^***^	0.002
High	N	High – Low	8.95	3.94	2.27^*^	0.023
	Y	High – Low	−8.59	2.14	−4.02^***^	< 0.001

^*^p < 0.05, ^**^p < 0.01, ^***^p < 0.001.

N, isolated condition. Y, contextual condition.

SE, standard error; *df*, degree of freedom.

High, Mean + *1SD*. Low, Mean-*1SD*.

### 4.3. Results of the reaction times data

Only correct responses to Yes trials were included in the analysis. Using the AIC, familiarity, English vocabulary proficiency, context, and first-order interaction of English vocabulary proficiency and context were selected as fixed effects, and participants and items were selected as random effects in the model. The results for the reaction times are shown in Table 6. The main effect of familiarity was significant (*F* (1, 45.44) = 38.47, *p* < 0.001), indicating that participants responded faster with increasing levels of familiarity. The main effect of English vocabulary proficiency was non-significant (*F* (1, 29.01) = 0.05, *p* = 0.816). Similarly, the main effect of context did not reach statistical significance (*F* (1, 41.07) = 2.26, *p* = 0.141). However, a significant interaction was observed between English vocabulary proficiency and context (*F* (1, 1108.40) = 4.01, *p* = 0.046).

**Table 6:** Results of LME model analysis of reaction times.

Variables	Estimate	SE	*df*	*t*	*pr* (*>|t|*)
Intercept	3.16	0.02	43.41	167.92^***^	<0.001
Familiarity	−0.06	0.01	45.44	−6.20^***^	<0.001
English Vocabulary Proficiency	0.00	0.02	29.01	0.23	0.816
ContextY	−0.03	0.02	41.07	−1.50	0.141
English Vocabulary Proficiency: ContextY	0.02	0.01	1108.40	2.00^*^	0.046

^*^p < 0.05, ^***^p < 0.001.

Participants = 31. Items = 44. Total observation = 1181.

SE, standard error. *df*, degree of freedom.

The optimal model is *lmer* [logrt ~ Vocabulary Proficiency + Context + Familiarity + Vocabulary Proficiency: Familiarity + (1|item) + (1|participant), data = data, lmerControl optimizer = “bobyqa,” optCtrl = list (maxfun = 100000)].

Table 7 presents results from the simple main effect tests. English vocabulary proficiency had no significant effect whether or not there was context [*t* (32.17) = 0.22, *p* = 0.831; *t* (32.05) = 0.67, *p* = 0.506]. For learners with high English vocabulary proficiency, there was no significant difference between the contextual and isolated conditions [*t* (55.41) = 0.65, *p* = 0.519]. However, for learners with low English vocabulary proficiency, the reaction time under the contextual condition was significantly shorter than that under the isolated condition [*t* (55.20) = 2.14, *p* = 0.037].

**Table 7:** Results of simple main effects of context and English vocabulary proficiency.

Moderator Levels	Contrast	Estimate	SE	*df*	*t*	*pr* (*>|t|*)
N	High – Low	−0.00	0.02	32.17	−0.22	0.831
Y	High – Low	0.01	0.02	32.05	0.67	0.506
Low	N – Y	0.04	0.02	55.20	2.14^*^	0.037
High	N – Y	0.01	0.02	55.41	0.65	0.519

* *p* < 0.05.

N, isolated condition. Y, contextual condition.

SE, standard error; *df*, degree of freedom.

High, Mean + *1SD*. Low, Mean-*1SD*.

## 5. Discussion

While several studies on the processing of Japanese loanwords exist (e.g.,Yamato and Tamaoka, 2013; Tamaoka, 2018), they have often focused only on a limited range of variables. Moreover, there has been a lack of systematic exploration of the processing of loanwords by advanced Chinese JFL learners. Consequently, this study investigates the influence of English-Japanese phonological similarity, familiarity, context, and English vocabulary proficiency on the processing of Japanese loanwords in advanced Chinese JFL learners. Furthermore, this study develops a loanword processing model specifically tailored to advanced Chinese JFL learners. The results will serve as a useful reference for the acquisition of Japanese loanwords.

### 5.1. The influencing factors of loanword processing

Based on accuracy rates, phonological similarity had a significant influence on loanword processing, enhancing a learner’s ability to accurately understand loanwords. However, we found no significant impact on reaction time in contrast with previous research results (Yamato and Tamaoka, 2013; Tamaoka, 2018). It was formulated the hypothesis that the phonological similarity would likely be facilitating the processing of Japanese loanwords. Consequently, the aforementioned results partly align with Hypothesis 1. Here, we discuss why no significant impacts existed in terms of reaction time. Advanced learners, due to their high level of Japanese proficiency, rely less on the phonetic representation of English than intermediate learners. Perhaps they have reached a stage where Japanese lexical processing is highly automated, enabling them to access conceptual representations directly without relying too much on English as an intermediary step. To fully understand how learner reliance on L2 and L3 processing evolves during different stages of acquisition, future research should include discussions on elementary and intermediate learners.

Regarding familiarity, in terms of both accuracy rates and reaction times, our findings indicate significant effects of familiarity on loanword processing. Higher levels of familiarity with loanwords were associated with higher accuracy rates and shorter reaction times. This suggests that familiarity plays a crucial role in loanword processing, even for advanced learners. These results, supporting Hypothesis 2, align with previous studies (Yamato et al., 2010; Ang et al., 2016; Li et al., 2020) and emphasize the stable facilitating effect of familiarity on loanword processing across different language pairs.

In terms of context, despite the lack of significant main effects, interactions between context and other factors existed. The results of the simple main effects analysis indicate that the presence of context did not always facilitate the processing of loanwords, thus providing insufficient evidence to support Hypothesis 3. This finding is inconsistent with previous results (e.g., Sereno et al., 2003; Goldwater et al., 2009). An interaction between context and phonological similarity was observed in terms of accuracy rates. This indicates that, when phonological similarity was low, context served as a compensatory and facilitating factor. Nevertheless, when phonological similarity was high, the presence of context resulted in an inhibitory effect. This may have been because, under conditions of high phonological similarity, learners partially relied on their L2 English vocabulary to process the loanwords. Therefore, excessive contextual information can increase learner language processing load, leading to an inhibitory effect.

The analysis of the second-order interaction revealed that the inhibitory effect of context was strongest when both phonological similarity and familiarity were high. This suggests that, when both phonological similarity and familiarity are high, perhaps learner processing of loanwords reaches a high level of automatization. Under such circumstances, the presence of context may introduce a certain degree of interference in learner processing, thereby reducing accuracy rates. Additionally, when both phonological similarity and familiarity are low, although the accuracy rate under the contextual condition is relatively low, it does not reach a significant level. Finally, the analysis of reaction times indicates that when learner English vocabulary proficiency is low, context facilitates the rapid processing of loanwords. However, for learners with high vocabulary proficiency, the facilitating effect of context is not significant. These results indicate that the impact of context on loanword processing depends on the specific characteristics of the loanwords (i.e., English-Japanese phonological similarity and familiarity with loanwords) and learner language proficiency (i.e., the proficiency of English and Japanese). The interaction between context and other factors in the processing of loanwords in advanced Chinese JFL learners will be further discussed in Section 5.2.

Lastly, there were no significant differences in English vocabulary proficiency in terms of accuracy rates and reaction times, which does not support Hypothesis 4. This finding is inconsistent with the results of previous studies (Tamaoka, 1997; Yamato and Tamaoka, 2013). There are two possible reasons for this result: (1) all participants in this study were advanced learners of Japanese, and the self-evaluation results of participant language proficiency indicated that their L3 Japanese proficiency was significantly higher than their L2 English proficiency. The frequency of L3 Japanese use was also significantly higher than that of L2 English use. This suggests that, for advanced learners, the impact of L2 English proficiency on the processing of loanwords in L3 Japanese among Chinese JFL learners is limited. (2) Although the influence of English vocabulary proficiency was not significant, as mentioned earlier, the influence of phonological similarity between Japanese and English still existed. We speculate that this might be related to the methodology used for testing English vocabulary proficiency in this study. The assessment of English vocabulary proficiency in this study employed a visual presentation method. Further investigation is needed to examine whether the processing of L3 Japanese loanwords among advanced Chinese JFL learners would be influenced by English vocabulary proficiency if an auditory presentation method were used.

### 5.2. The model of loanword processing in advanced Chinese JFL learners

**Figure 3:**
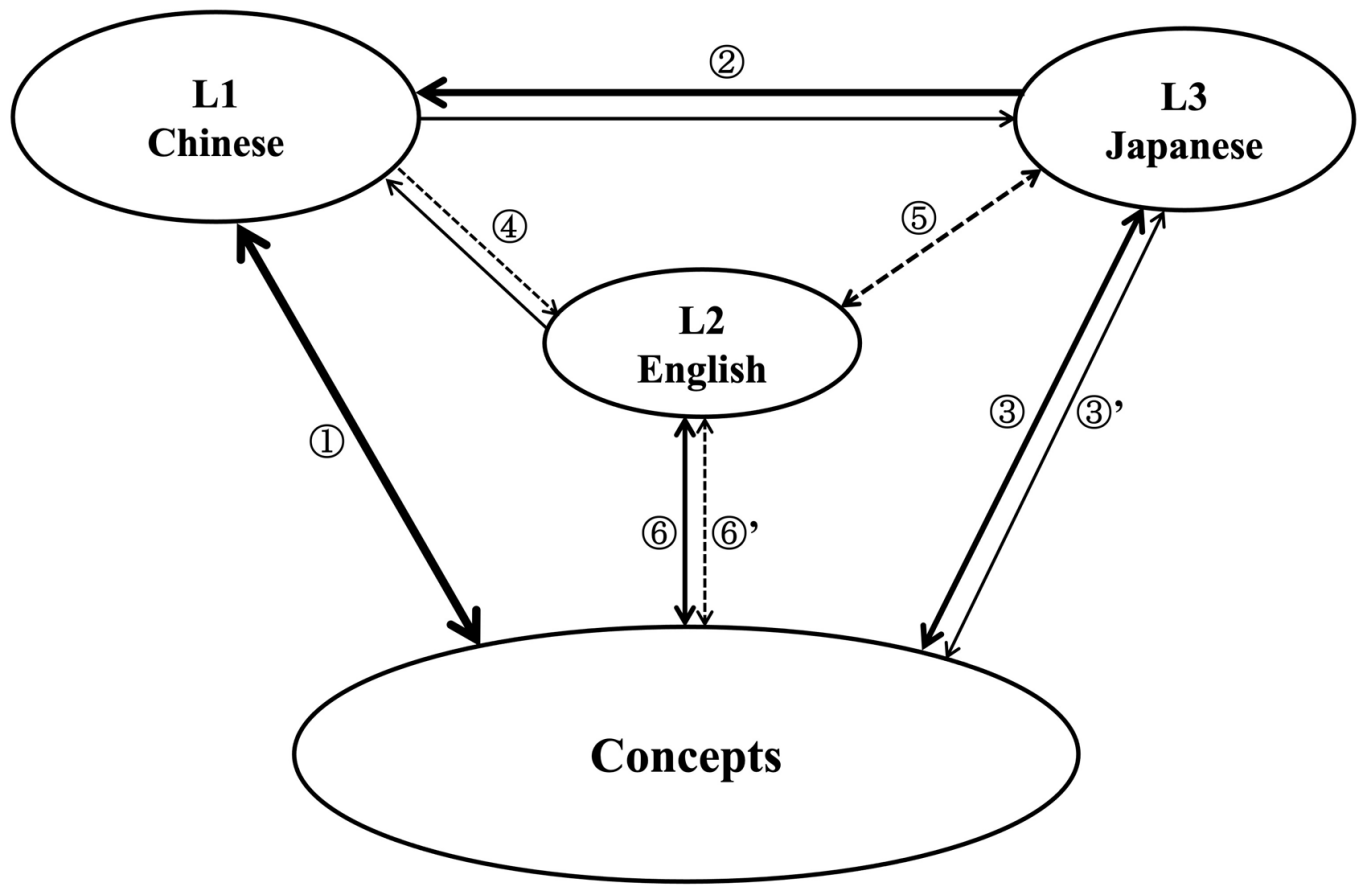
Processing of Japanese loanwords by advanced Chinese JFL learners under isolated and contextual conditions.

As mentioned earlier, there is a lack of study on the processing model of Japanese loanwords by Chinese JFL learners, especially in terms of multiple perspectives and factors. Therefore, in this section, a processing model for loanwords used by advanced Chinese JFL learners is constructed. The preceding discussion shows that familiarity has the most significant impact among the four factors examined. In contrast, the effects of other factors are often limited and interconnected, exerting themselves collectively. Therefore, we propose that familiarity with Japanese loanwords should be considered the primary factor in loanword processing. Contextual information then follows, whereas phonological similarity and English vocabulary proficiency have a minor influence. To construct a processing model for advanced Chinese JFL learners, we developed a revised hierarchical model (Figure 3) based on previous studies (Kroll and Steward, 1994) and the aforementioned analysis.

According to the self-evaluation results for participant language proficiency and the lexical processing model used in previous studies (Chen and Leung, 1989; Kroll and Steward, 1994), learners can be characterized as unbalanced trilinguals, with Chinese, Japanese, and English ranked from highest to lowest in terms of proficiency. In Figure 3, lines ①–⑥ represent the links between a lexical representation and conceptual representation of Chinese, Japanese, and English. The thickness of a line indicates the strength of a link. During processing in advanced Chinese JFL learners for the acquisition of L3 Japanese, the paths through the lexical link (lines ② → ①) or the conceptual link (line ③) are the most significant. On the contrary, the processing path through the L2 English lexical representation (lines ⑤ → ⑥) may not be the dominant pathway. Additionally, a processing path via Chinese and English (lines ② → ④ → ⑥) is unlikely to exist. Research on the English lexical processing of Chinese English learners indicates that, as learner proficiency improves, the lexical processing mechanism transitions from a lexical link to a conceptual link (Chen and Leung, 1989). Considering the overwhelming influence of familiarity and the relatively weak correlation between Japanese loanwords and Chinese lexical representation, it is believed that, from an overall perspective, advanced Chinese JFL learners tend to directly access L2 Japanese loanwords using conceptual representation (line ③). However, a link is also influenced by other factors, such as phonological similarity, context, and English vocabulary proficiency.

The analysis of accuracy rates reveals that when there is low phonological similarity and high familiarity, context promotes learners’ correct judgments. This means that, due to the presence of context, learners activate the conceptual representation in advance with the assistance of context. As a result of this activation, the three lexical representations of Chinese (line ①), Japanese (line ③), and English (line ⑥) are also activated. Since the experimental stimuli are in Japanese with low phonological similarity to English, the activation of English lexical representations should be the weakest (i.e., line ⑥’). However, when both phonological similarity and familiarity are high, the context reduces the accuracy rate. This indicates that with the activation of English lexical representation (line ⑥), due to the high phonological similarity, where the English lexical representation that was originally not involved in the bilingual competition, is now included in the competition, resulting in the emergence of trilingual competition. In this kind of situation, if learners are unable to effectively utilize the activated English representations, their processing accuracy decreases. On the other hand, the reason for the disappearance of a significant effect from the context when both phonological similarity and familiarity are low, may be because that now it is highly challenging for learners, even with the activation of conceptual representation facilitated by the presence of context, to give correct responses. Moreover, although the context does not now exert a significant impact on the accuracy rate, the accuracy rate under the contextual condition is relatively low. This could be due to the increased cognitive load imposed on learners when processing contextual information, leading to a decrease in accuracy rate. In summary, when both phonological similarity and familiarity are high, the presence of context inhibits performance due to trilingual competition. In contrast, when both phonological similarity and familiarity are low, the lack of significant difference with context is possibly due to the additional cognitive load imposed by the context on learners in an already challenging situation (i.e., low familiarity and low phonological similarity), which results in a negative impact.

The results for reaction time show that the interaction between context and English vocabulary proficiency is significant. Context has a significant facilitating effect on learners with low proficiency but not on learners with high proficiency. Therefore, in the absence of context, the processing path for learners with low proficiency is represented by line ③’, but with context, it transitions from line ③’ to line ③, gradually forming a direct connection in a rapid response model. Therefore, context has the potential to assist in rapid judgment when English proficiency is low. However, if there is significant competition between the three languages (Chinese, English, and Japanese), it can result in a decrease in processing efficiency. In such a case, the presence of context alone may not be sufficient to overcome the challenges posed by language competition to ensure accurate processing. Therefore, while context can be beneficial in certain scenarios, it is important to mitigate potential interference from the simultaneous activation of multiple languages to maintain processing accuracy and efficiency.

### 5.3. Suggestions on Chinese JFL learners’ acquisition of Japanese loanwords

Familiarity significantly influences the processing of loanwords. Previous studies on Japanese language education have indicated that Chinese JFL learners, especially beginners or intermediate learners, tend to avoid using loanwords (Deng, 2018). This implies that Chinese JFL learners, influenced by their L1, have a tendency to prefer using *Kanji* words over loanwords when encountering Japanese lexicons with the same meaning that include both options. The results of the current study revealed that even advanced JFL learners exhibited an average reaction time of 1548.22 ms for all loanwords, indicating room for improvement in their response speed. Hence, it is crucial for learners to actively engage with loanwords, gradually enhancing their familiarity with them.

The results indicate that English-Japanese phonological similarity and English vocabulary proficiency have a weak influence on the processing of loanwords in advanced JFL learners. This suggests that the link between Japanese lexical representation and English lexical representation is weak for Chinese learners. If learners cannot handle the bilingual competition between English and Japanese well, it decreases the accuracy and speed with which they process Japanese loanwords. Therefore, to maximize the benefits of learners’ existing English knowledge, it is recommended that they focus on word-pair learning between English and Japanese when teaching and learning loanwords. Because of the competition between the three languages, it is advised that paired learning between English and Japanese be used. This approach enhances bilingual coordination and mitigates the negative impact of competition.

We observed that context does not always have a positive impact on advanced Chinese JFL learners and can even have an inhibitory effect. Therefore, when teaching Japanese, it is crucial to carefully consider the characteristics of the context and select appropriate materials that facilitate learner acquisition. Based on the findings of this study, it is recommended that teachers analyze the specific characteristics of loanwords, make thoughtful adjustments to the contextual materials, and implement targeted teaching strategies to cater to the needs of advanced Chinese JFL learners.

## 6. Conclusion

In this study, a lexical decision task was utilized to examine how English-Japanese phonological similarity, familiarity, context, and English vocabulary proficiency impacted the processing of Japanese loanwords among Chinese JFL learners. An analysis using a (generalized) linear mixed-effect model showed that the influence of English-Japanese phonological similarity, English vocabulary proficiency, and context on Japanese loanword processing was not always positive. As learners’ Japanese proficiency improved, they tended to process Japanese loanwords directly based on conceptual representations, with English vocabulary proficiency showing no significant influence. When both Japanese familiarity and English-Japanese phonological similarity were high, context exerted an inhibitory effect. These conclusions underscored the complexity of examining the lexical processing mechanism in trilingual individuals and emphasized the need to consider various influencing factors when investigating the associations between representations.

This study has several limitations. The participants consisted solely of advanced Chinese JFL learners, which may reduce the generalizability of the findings when considering Chinese JFL learners at all proficiency levels. Consequently, it would be valuable to expand the scope of participants to include elementary and intermediate Chinese JFL learners and compare the results with the findings of the current study, to further investigate how the processing of Japanese loanwords by Chinese JFL learners changes as their Japanese proficiency improves. Additionally, as a special component of Japanese vocabulary, to more deeply understand how these factors examined in this study play their roles, further exploration of the processing of Japanese loanwords by English JFL learners and Japanese EFL learners would present an intriguing avenue for further investigation. By comparing the findings of such a study with the current research, we can gain additional insights into the processing mechanism of Japanese loanwords.

## Data availability statement

The raw data supporting the conclusions of this article will be made available by the authors, without undue reservation.

## Ethics statement

The studies involving humans were approved by the Beijing Center for Japanese Studies, Beijing Foreign Studies University. The studies were conducted in accordance with the local legislation and institutional requirements. The participants provided their written informed consent to participate in this study.

## Author contributions

YG, QS, and XF contributed to the conception of the work and revised the manuscript and confirmed its final version. YG and XF collected the experimental data. QS conducted the data analysis. YG and QS wrote the first manuscript. All authors contributed to the article and approved the submitted version.

## Funding

This study was supported by the “Excellent Talents Program of Beijing Foreign Studies University 2021 (2021-ZYQNJS-011).

## Conflict of interest

The authors declare that the research was conducted in the absence of any commercial or financial relationships that could be construed as a potential conflict of interest.

## Publisher’s note

All claims expressed in this article are solely those of the authors and do not necessarily represent those of their affiliated organizations, or those of the publisher, the editors and the reviewers. Any product that may be evaluated in this article, or claim that may be made by its manufacturer, is not guaranteed or endorsed by the publisher.

## Footnotes

^1^https://chuta.cegloc.tsukuba.ac.jp/
