# Supplementary material for: Factors in cognitive processing of Japanese loanwords by advanced Chinese Japanese-as-a-foreign-language learners
Source: Front Psychol. 2023 Aug 17;14:1224830. doi: 10.3389/fpsyg.2023.1224830 (PMC10469616; doi:10.3389/fpsyg.2023.1224830)
Supplement: Supplementary file 1 [file Data_Sheet_1.pdf]

Supplementary Materials

Table 1 Loanwords of the Experiment.

**Loanwords of isolated condition**		**Loanwords of contextual condition**	
プラス<br>(plus)	ロケット<br>(rocket)	タイプ<br>(type)	リボン<br>(ribbon)
ナイロン<br>(nylon)	テキスト<br>(text)	ケース<br>(case)	ブレーキ<br>(brake)
ホーム<br>(home)	リポート<br>(report)	チャンス<br>(chance)	オートバイ<br>(autobicycle)
ミス<br>(miss)	レジャー<br>(leisure)	ルーズ<br>(loose)	ラケット<br>(racket)
クラス<br>(class)	サークル<br>(circle)	バケツ<br>(bucket)	アイロン<br>(iron)
ハンサム<br>(handsome)	エキスパート<br>(expert)	スクール<br>(school)	トンネル<br>(tunnel)
ナイフ<br>(knife)	トラック<br>(truck)	ハイキング<br>(hiking)	ダイヤ<br>(diagram)
チェック<br>(check)	ベテラン<br>(veteran)	チーム<br>(team)	レジ<br>(register)
オーソドックス<br>(orthodox)	デモ<br>(demonstration)	アンテナ<br>(antenna)	セーター<br>(sweater)
スタイル<br>(style)	エアコン<br>(air conditioner)	コピー<br>(copy)	ライバル<br>(rival)
サンドイッチ<br>(sandwich)	ワープロ<br>(word processor)	ソフト<br>(soft)	ビル<br>(building)

Context

恋人なら、どんなタイプの人が好きですか？
(What type of person would you like as a lover?)

プレゼントをもらったら、すぐにケースを外すのがよくない。
(When you receive a present, it's not a good idea to immediately take it out of the case.)

今度から気を付けるから、もう一度チャンスをください。
(I'll be careful from now on, so please give me another chance.)

彼は友達には厳しいけど自分にはルーズです。
(He is strict with his friends, but loose with himself.)

花屋に水の入ったバケツがいくつか並んでいます。
(There are several buckets filled with water lined up at the flower shop.)

彼女は昔、英語スクールに通っていたため、英語がうまいです。
(Since she used to attend an English school in the past, she is good at English.)

天気がいいから、どこかの山へハイキングしましょう。
(The weather is nice, so let's go hiking to some mountain.)

今日の試合はアメリカとイギリスの二つのチームが出る。
(Today's match features two teams, one from America and the other from the United Kingdom.)

台風のせいでテレビのアンテナが折れてしまいました。
(Due to the typhoon, the TV antenna got broken.)

今日は 5 人の学生が授業に出るので、資料を 5 人分にコピーしてください。
(Please copy the materials for the five students who will attend the class today.)

一番好きなタオルを買うのなら、もちろんソフトなのが肌にいいです。
(If you're going to buy your favorite towel, of course, a soft one is good for your skin.)

誕生日プレゼントの箱に、きれいなリボンがついています。
(The birthday present box has a beautiful ribbon attached to it.)

運転するとき急に人が現れて、急ブレーキをかけました。
(While driving, a person suddenly appeared, so I hit the brakes hard.)

東南アジアの国の街では、オートバイに乗る人をよく見かけます。
(In the cities of Southeast Asian countries, you often see many people riding autobicycles.)

テニスやバドミントンをやるには、ラケットが必要です。
(To play tennis or badminton, you need a racket.)

彼は毎日、次の日に着るシャツにアイロンをかけています。
(He irons the shirt he will wear the next day every day.)

汽車がトンネルに入ると真っ暗になりました。
(When the train entered the tunnel, it became pitch dark.)

駅の壁には地下鉄のダイヤが貼られています。
(The subway diagram is posted on the walls of the station.)

お金はそこにあるレジで払って下さい。
(Please make the payment at the register over there.)

寒くなってきたので、セーターを着てきました。
(Since it’s getting cold, I wore a sweater.)

真面目に勉強しているのは、ライバルに負けたくないからです。
(The reason I’m studying seriously is that I don’t want to lose to my rivals.)

さすが大都市、高いビルがたくさん並んでいます。
(As expected of a big city, there are many tall buildings lined up.)

Table 2 Fillers of the Experiment.

Fillers of isolated condition		Fillers of contextual condition	
プロセース	ヲテレクマ	バイオリーン	サドババハヤ
ポスット	スセルトレ	コンピューッター	イカドラベ
レポット	ツガレドハ	デッタベース	エカサンマ
プラーン	ドレツミロ	キーワド	スグレレファ
シャーター	ハラコクレ	フォック	カニカレド
スクリン	インガフラセ	プレゼーント	イケンドチル
コレクッション	エレカヅラ	カッテン	ツラロテ

Context

彼女はバイオリーンを弾くのが好きです。
(She enjoys playing the バイオリーン.)

明日が締め切りなのに、今日急にコンピューッターが壊れて困っている。
(Tomorrow is the deadline, but today, the コンピューッター suddenly broke down, and I’m in trouble.)

このデッタベースにはものすごい量の情報が入っている。
(This デッタベース contains an enormous amount of information.)

文章を読む前に先にキーワドを見ることで、内容理解が促進される。
(Looking at キーワド before reading the text can enhance understanding of the content.)

洋食を食べるときよく使うのがナイフとフォックです。
(When eating Western food, knives and フォック are commonly used.)

誕生日プレゼーントをたくさんもらったのでうれしい。
(I am happy because I received a lot of birthday プレゼーント.)

寝る前に、必ずカッテンを閉めてください。
(Before going to sleep, please make sure to close the カッテン without fail.)

王さんはサドバハヤな人間ですので、嫌われている。
(Since Mr. Wang is a サドバハヤ person, he is disliked.)

天気のいい日にイカドラベに行きたい。
(I want to go イカドラベ on a sunny day.)

週末にエカサンマをやったりして過ごす。
(I spend the weekend doing things like エカサンマ or other leisure activities.)

先生は授業で僕がスグレレフアな人だと言って褒めてくれた。
(The teacher praised me in class, saying that I am an スグレレフア student.)

今回の地震はカニカレド3 だったそうです。
(It seems that this earthquake was classified as a カニカレド 3.)

本を読むことが私たちのイケンドチルにいいのです。
(Reading books is good for our イケンドチル .)

途中から離れるときに、ツラロテをしてください。
(Please make sure to ツラロテ when you leave midway.)
